# The Hospital Almoners' Council

**Published:** 1912-12-14

**Authors:** 


### The Hospital Almoners' Council.

The annual meeting of the Hospital Almoners' Council took place at Caxton Hall on Friday, December 6, Sir James Goodhart, Bart. (Vice-President), in the chair. The Report draws attention to the fact that the Council now numbers among its members many representatives of London and provincial hospitals. During the past year almoners trained by the Council have been appointed at two London hospitals—the Royal Waterloo Hospital for Children and Women, and the General Lying-in Hospital, York Road—while others have secured posts as assistants. Two candidates are being specially trained for posts in the North of England, and nine others are now in training. The Chairman, in moving the adoption of the Report, drew attention to the work of the Council in stimulating a demand which it is now called upon to supply, and made a reference to the importance of the recommendations of the Special Committee of King Edward's Hospital Fund as they affect the future of the movement. Particular attention was drawn by various speakers to the necessity of definite training, and to the danger of the appointment of those who are unable to benefit by the experience of their predecessors. The need for this training had been realised by those hospitals which had sent almoners to be trained by the Council whom they had appointed and who had already begun work. Certificates have now been granted to twenty-one almoners, who have qualified for this work by training under the Council since its establishment as an independent body in November 1907.

